# Tryptophan depletion affects compulsive behaviour in rats: strain dependent effects and associated neuromechanisms

**DOI:** 10.1007/s00213-017-4561-5

**Published:** 2017-03-09

**Authors:** A. Merchán, S. V. Navarro, A. B. Klein, S. Aznar, L. Campa, C. Suñol, M. Moreno, P. Flores

**Affiliations:** 1 Department of Psychology, University of Almería, CeiA3, Carretera de Sacramento s/n, 04120 Almería, Spain; 2 Department of Drug Design and Pharmacology, University of Copenhagen, 2100 Copenhagen, Denmark; 3 Research Laboratory for Stereology and Neuroscience, Bispebjerg and Frederiksberg Hospitals, Copenhagen University Hospital, 2300 Copenhagen, Denmark; 4 Institute of Biomedical Research of Barcelona, IIBB-CSIC-IDIBAPS, CIBERESP (CS), CIBERSAM (LC), 08028 Barcelona, Spain

**Keywords:** Compulsivity, Inhibitory control, Chronic tryptophan depletion, Schedule-induced polydipsia, Monoamines, 5-HT_2A_ binding

## Abstract

**Rationale:**

Compulsive behaviour, present in different psychiatric disorders, such as obsessive-compulsive disorder, schizophrenia and drug abuse, is associated with altered levels of monoamines, particularly serotonin (5-hydroxytryptamine) and its receptor system.

**Objectives:**

The present study investigated whether 5-HT manipulation, through a tryptophan (TRP) depletion by diet in Wistar and Lister Hooded rats, modulates compulsive drinking in schedule-induced polydipsia (SIP) and locomotor activity in the open-field test. The levels of dopamine, noradrenaline, serotonin and its metabolite were evaluated, as well as the 5-HT_2A_ and 5-HT_1A_ receptor binding, in different brain regions.

**Methods:**

Wistar rats were selected as high (HD) or low (LD) drinkers according to their SIP behaviour, while Lister hooded rats did not show SIP acquisition. Both strains were fed for 14 days with either a TRP-free diet (T−) or a TRP-supplemented diet (T+)

**Results:**

The TRP depletion diet effectively reduced 5-HT levels in the frontal cortex, amygdala and hippocampus in both strains of rats. The TRP-depleted HD Wistar rats were more sensitive to 5-HT manipulation, exhibiting more licks on SIP than did the non-depleted HD Wistar rats, while the LD Wistar and the Lister Hooded rats did not exhibit differences in SIP. In contrast, the TRP-depleted Lister Hooded rats increased locomotor activity compared to the non-depleted rats, while no differences were found in the Wistar rats. Serotonin 2A receptor binding in the striatum was significantly reduced in the TRP-depleted HD Wistar rats.

**Conclusions:**

These results suggest that alterations of the serotonergic system could be involved in compulsive behaviour in vulnerable populations.

## Introduction

Compulsivity represents the performance of repetitive and functionally impairing overt or covert behaviours without adaptive function, performed in a habitual or stereotyped fashion, either according to rigid rules or as a means of avoiding perceived negative consequences (Fineberg et al. 2014). Neuropsychiatric disorders characterized by compulsivity are included in the newly created Diagnostic and Statistical Manual of Mental Disorders, fifth edition (DSM-5) “obsessive-compulsive and related disorders” (OCRDs) cluster, such as obsessive-compulsive disorder (OCD), body dysmorphic disorder, trichotillomania (repetitive hair pulling), hoarding disorder and excoriation (skin-picking) (American Psychiatric Association 2013). Moreover, this behaviour is also present across different disorders, such as schizophrenia, attention-deficit hyperactivity disorder (ADHD), pathological gambling, eating disorders, depression or substance addiction (Skodol and Oldham 1996). Dysfunctions in cortico-limbic-striatal circuits, involving areas such as mOFC, caudate-putamen, amygdala and hippocampus, have been associated with the symptomatology in OCD (Gillan and Robbins 2014; Rǎdulescu and Marra 2016).

Evidence from animal and human studies implicates the serotonin 5-hydroxytryptamine (5-HT) system in impulsivity and compulsivity (Eagle and Baunez 2010; Fineberg et al. 2010). Mice lacking the gene encoding brain tryptophan hydroxylase 2 (Tph2−/−), the initial and rate-limiting enzyme in 5-HT synthesis, showed intense impulsive and compulsive behaviours to include extreme aggression (Angoa-Pérez et al. 2012). Moreover, studies on 5-HT depletion by excitotoxic lesions in rats have revealed an increase of perseverative responding in the five-choice serial reaction time task (Winstanley et al. 2004), impairment of behavioural flexibility measured through the reversal learning task (Bari et al. 2010; Lapiz-Bluhm et al. 2009; Wallace et al. 2014) and an increment of compulsive cocaine seeking under punishment (Pelloux et al. 2012).

A non-invasive and more naturalistic method to reduce central 5-HT is through nutritional depletion of the 5-HT precursor tryptophan (TRP). Under normal physiological conditions, the biosynthesis of 5-HT is limited by the availability of the essential amino acid TRP (Fernstrom 1983; Gessa et al. 1974). Rats receiving a TRP-free diet reduced the 5-HT synthesis, content (Gessa et al. 1974) and release (Stancampiano et al. 1997a, b). While acute tryptophan depletion by diet (ATD) produced a moderate serotonergic reduction in adult rats (Brown et al. 1998; Lieben et al. 2004), chronic tryptophan depletions (CTD) have shown stronger effects, reducing 5-HT brain levels to 35–40% at 14 days (Fadda et al. 2000) and to 75% at 5-week exposures (Vergnes and Kempf 1981). Moreover, long-term TRP-depleting diets lead to changes in serotonergic receptors in animals, increasing serotonin 5-HT_2A_ receptor density but having no effect on serotonin 5-HT_1A_ receptor (Cahir et al. 2007; Franklin et al. 1999). Behavioural studies in rodents have demonstrated that a TRP depletion by diet increased aggressiveness (Vergnes and Kempf 1981), locomotor activity (Vergnes and Kempf 1981) and sexual behaviour (Fratta et al. 1977). The increase of these behaviours may suggest a lack of inhibitory control leading to compulsive behaviour. However, only one study has evaluated the effect of acute TRP depletion on some facets of compulsivity, showing no effects on behavioural flexibility in reversal learning or in an extinction paradigm (Van der Plasse and Feenstra 2008). Thus, the effects of chronic TRP depletion by diet on compulsivity remain unknown.

Because of its characteristics of “excessiveness” and “persistence”, schedule-induced polydipsia (SIP) is a useful model to study neuropsychiatric disorders characterized by the presence of compulsive behaviour (Ford 2014; Gilpin et al. 2008; Hawken et al. 2011; Hawken and Beninger 2014; for review, see Moreno and Flores 2012). The SIP model is characterized by the development of excessive drinking in food-deprived animals exposed to intermittent food reinforcement schedules (Falk 1961, 1971). Important differences between individuals in the amount of fluid intake and licks support the differentiation of two phenotypes of rats, one with high or excessive drinking (HD) and a second group with low or not SIP acquisition (LD) (López-Grancha et al. 2008). Recent data have shown that HD animals present increased levels of 5-HT and metabolites in the amygdala compared to the LD group (Moreno et al. 2012). In addiction, the systemic administration of citalopram and the serotonin 5-HT_2A/C_ receptor agonist 2,5-dimethoxy-4-iodoamphetamine (DOI) reduced dose-dependent compulsive drinking in HD rats, without affecting the drinking behaviour of LD rats (Navarro et al. 2015). All those data point out the involvement of 5-HT system in the vulnerability to the development of compulsive drinking between HD and LD rats.

In our laboratory, Wistar rats are commonly used in SIP, showing individual differences between HD and LD phenotypes. However, no previous studies have tested the strain-dependent differences between Wistar and Lister Hooded rats in SIP acquisition. In fact, behavioural differences in inhibitory control have been observed between these two strains of rats. Wistar rats compared to Lister Hooded rats show more anticipatory responses in a three-choice serial reaction time task and more food hoarding behaviour (Broersen and Uylings 1999), indicating a poorer inhibitory control of this strain and a higher vulnerability to develop compulsive drinking on SIP.

We hypothesised that a reduction of 5-HT through a chronic TRP depletion by diet will increase the compulsive behaviour in vulnerable populations such as the HD Wistar rats compared to non-vulnerable populations such as LD Wistar rats and Lister Hooded rats and that could be accompanied by changes in the serotonin 5-HT_2A_ receptor, a serotonin receptor subtype proposed as a candidate for mediating compulsive behaviour (Aznar and Hervig 2016; Aznar and Klein 2013; Fineberg et al. 2010, 2011; Navarro et al. 2015). To test the previous hypothesis, we screened high and low drinking rates during SIP in both strains. Next, we produced a dysfunction of the 5-HT system in the brain by a chronic TRP depletion by diet and evaluated possible motor disruptions or hyperactivity in an open-field test and the effect on compulsive drinking on SIP. Then, brain monoamine levels and serotonin 5-HT_2A_ and 5-HT_1A_ receptor densities were measured in different brain regions of the cortico-limbic-striatal circuits associated with compulsivity.

## Methods

### Subjects

Two strains of rats were used for this experiment: adult male Lister Hooded rats from Charles River (Barcelona, Spain) and adult male Wistar rats from Harlan Iberica (Barcelona, Spain).

Both strains of rats weighed approximately 300–400 g at the beginning of the experiment. The animals were housed three/cage or two/cage (57 × 35 × 20 cm) at 22 °C with 08:00–20:00-h light-dark cycle, with food and water available ad libitum. Before the SIP training and after 10 days of habituation to the vivarium conditions, the rats were weighed and handled daily. They were gradually reduced to 80–85% of their free-feeding body weight by controlled feeding and then maintained at this level of deprivation throughout the experiment. Food was made available by daily feeding of lab chow approximately 30 min after each experimental session. Water was always available in the home cages.

Rats were assigned to each experimental group taking into consideration the amount of water consumed in the previous experimental SIP, in order to match all groups. The Wistar rats were split as following: high drinkers receiving a TRP-free diet (*n* = 7), high drinkers receiving a control diet (*n* = 7), low drinkers with a TRP-free diet (*n* = 7) and low drinkers with a control diet (*n* = 7). The Lister Hooded rats, as they did not show differences in SIP (water intake and licks), were divided into two groups depending on the diet: One group (*n* = 8) received a TRP-free diet, while the other group (*n* = 9) received a control diet. Once the animals started the specific diets, they were housed in cages individually (50 × 25 × 18 cm) to control body weight.

All procedures were conducted in accordance with the Spanish Royal Decree 53/2013 on the protection of experimental animals, with the European Community Council Directives (86/609/EEC) and with the University of Almería Animal Research Committee approval.

### Experimental design

**Fig. 1:**
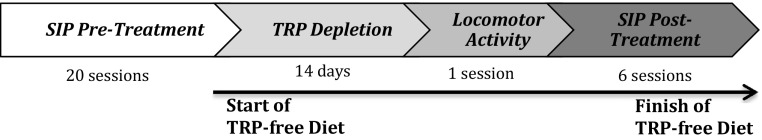
Experimental procedure illustrated in a timetable

The experiment was developed in two phases. One previous phase consisted of screening the acquisition of SIP in the Lister Hooded and Wistar rats. Once the rats were identified as high drinkers or low drinkers by their SIP behaviour, they were divided into groups depending on the given diet. After 14 days of TRP depletion by diet, the rats were exposed to different behavioural tasks. The order of presentation was as follows: one session of the open-field test and six sessions of SIP (see Fig. 1 for the entire experimental design).

### Apparatus and behavioural procedures

#### Schedule-induced polydipsia

We conducted the tests in ten standard operant conditioning chambers (MED Associates) that were 32 cm long × 25 cm wide × 34 cm high, with stainless steel grid floors. A detailed description of the apparatus has been provided previously for the SIP (López-Grancha et al. 2008; Moreno et al. 2012). The scheduling and recording of experimental events were controlled by a Med PC computer and commercial software (Cibertec SA, Spain).

##### Baseline consumption

All rats were individually housed in plastic cages containing a dish with the same amount of food to be delivered in the experimental sessions and the same water bottle used in the operant chambers. Over two successive days, 60 food pellets were placed together in a dish, and the amount of water consumed by each rat in 60 min was measured.

##### Magazine habituation

The day after the first baseline consumption sessions, rats were habituated to the test chambers for 60 min and were given 30 food pellets placed in the food magazine.

##### Schedule-induced polydipsia pre-treatment

After the magazine habituation, the animals were exposed to a fixed time 60-s (FT-60s) schedule of food pellet presentation during 60-min sessions. Water bottles containing 100 ml of fresh water were provided immediately before each session. After 20 daily sessions, the average water drinking for each animal was calculated based on the last three SIP sessions. Following Moreno et al.’s protocol (2012), rats are classified as high drinkers (HD) and low drinkers (LD) if their average water intake was above or below the group median, respectively. The following measures were recorded for each rat (a) total number of licks, (b) total amount of water (ml) removed from the bottle, (c) total number of magazine entries and (d) licking efficiency, which was calculated as the total number of licks/by the total solution consumed. Lick efficiency detects possible fine motor impairments or changes in the stereotypic manner of licking, which indicates with higher score values that the animal needs more total number of licks to obtain the same amount of target solution (Escher and Mittleman 2006).

##### Schedule-induced polydipsia post-treatment

After 14 days of the TRP depletion diet, the animals were exposed again to a FT-60s schedule of food pellet presentation during 60-min sessions. Water bottles with fresh water were available.

#### Spontaneous locomotor activity

The test was an open-field test, performed in eight Plexiglas activity cages (measuring 39 × 39 × 15 cm) equipped with photocell beams (16 × 16 × 16) interfaced to a microcomputer VersaMax Animal Activity Monitoring System (AccuScan Instruments Inc., USA). Spontaneous locomotor activity was measured as the number of photocell beam breaks due to the movements of the animals. TRP-depleted and TRP-non-depleted rats were tested for their locomotor responses to a novel environment in the activity cages. Rats were not habituated to the activity cages prior to this test. Spontaneous locomotor behaviour was quantified in 5-min blocks over a 60-min period following placement into the test cage. We measured total distance, counted as the number of centimetres travelled by the animal (an indicator of ambulatory activity).

### Tryptophan depletion diet

The TRP-free diet (TD08126, Harlan Laboratories S.A., Barcelona, Spain) had a standard nutritional value, but with a complete lack of TRP. The control groups were fed a similar diet, containing a standard amount of TRP (1.8 g/kg diet) (TD99366, Harlan Laboratories S.A., Barcelona Spain). A chronic TRP-free diet exposure of 14 days was given before the behavioural tasks as previous studies have established (Bortolato et al. 2008; Franklin et al. 2012; Stancampiano et al. 2013), and the amount of food was controlled in order to maintain the body weight at 80–85% of their free-feeding body weight.

### Brain analyses

The day after the SIP post-treatment, the rats were rapidly decapitated using a guillotine. Brains were quickly removed, frozen and stored at −80 °C. The cerebral hemispheres were separated, and each half was used either for measuring monoamines or for measuring serotonin receptor binding. The hemispheres were counterbalanced.

#### Brain monoamine analyses

For brain tissue preparation, the samples were thawed sufficiently to enable dissection of the prefrontal cortex, striatum, nucleus accumbens, hippocampus and amygdala (Moreno et al. 2012). These were weighed and homogenized in 0.4 N perchloric acid with 0.1 metabisulfite, 0.01% EDTA and 1 ng/ml cysteine. The homogenates were centrifuged at 15,000 rpm for 20 min at 4 °C, and supernatants were collected and frozen at −80 °C until biochemical analyses for determining the levels of norepinephrine (NE), dopamine (DA), serotonin (5-HT) and 5-hydroxy-3-indolacetic acid (5-HIAA), which were measured using reverse-phase high-performance liquid chromatography with electrochemical detection (+0.7 V). The mobile phase, containing 100 mM KH_2_PO_4_,0.1 mM Na_2_-EDTA, 2.06 mM PICB8 and 16% methanol, adjusted to pH 2.65 with orthophosphoric acid, was delivered at 1 ml/ min. Monoamines were separated on a 5-mm particle size column at 30 °C (Phenomenex C25 10 × 0.46 cm, Micron Analitica SA, Spain).

#### Autoradiography

To determine 5-HT_1A_R and 5-HT_2A_R binding in the Wistar and Lister Hooded rats, their brains were cut in 10-μm sections, mounted on Super Frost slides and stored at −80 °C. The protocol was modified from Klein et al. (2014). The 5-HT_2A_ autoradiography protocol was performed using ^3^H-MDL100907 [R(+)-α-(2,3-dimethoxyphenyl)-1-[2-(4-fluorphenyl)-ethyl]-4-piperidin-methanol] (specific activity, 2.8 TBq/mmol, Novandi Chemistry, Sweden; and non-specific binding was determined using 10 μM ketanserin tartrate (3-[2-[4-(4-fluorobenzoyl)-1-piperidinyl]-ethyl]-2,4[1H,3H] quinazolinedione tartrate) (Tocris Cookson Ltd., Bristol, UK). For 5-HT_1A_ autoradiography, we used ^3^H-WAY100635 (specific activity, 2.9 TBq/mmol, Novandi Chemistry) and measured non-specific binding with 10 μM 5-HT (Sigma–Aldrich, Copenhagen, Denmark). Briefly, the sections were allowed to thaw for 1 h at room temperature (RT) and then pre-incubated with 50 mM Tris–HCl (Sigma), pH 7.4, containing 0.01% ascorbic acid (Sigma) and 10 μM pargyline hydrochloride (*N*-methyl-*N*-2-propynylbenzylamine hydrochloride) (Research Biochemicals International, MA, USA) for 30 min at RT under constant gentle shaking. Sections were then incubated for 60 min at RT using the same buffer containing 2 nM of ^3^H-MDL100907 (1.5 nM of ^3^H-WAY100635 for 5-HT_1A_ binding). Following incubation, the slides were washed 2 × 2 min in ice-cold 50 mM Tris–HCl, pH 7.4, and 1 × 20 s in ice-cold dH_2_O and dried for 1 h under a gentle stream of air.

All sections were placed at 4 °C in a fixator containing paraformaldehyde vapour and later placed in an exicator box for 3 h before the slides, and the ^3^H-microscales (GE Healthcare, UK) were exposed to a BAS-TR2040 Imaging Plate (Science Imaging Scandinavia AB, Nacka, Sweden) for 3–14 days at 4 °C. Finally, the imaging plate was scanned on a CR-35 scanner (Raytest, Straubenhardt, Germany) and specific and non-specific binding was determined in the frontal cortex, striatum and hippocampus using the AIDA 5.0 software (Raytest) and expressed as femtomole per milligramme tissue equivalents (TE).

### Data analyses

Data analyses of the different strains were performed separately, due to the complexity of the groups. Analyses of variance (ANOVAs) were performed with two between-subject factors for the Wistar strain data, group (HD and LD) and treatment (T+ and T−) and one between-subject factor for the Lister Hooded strain data (treatment, T+ and T−). The within-subject factors were sessions of SIP, bins on the locomotor activity, body weight, brain monoamine data and receptor binding. Lick efficiency was analysed by repeated measures ANOVA, with treatment as between-subject and sessions as within-subject factors, in HD and LD rats. When appropriate, post hoc comparisons were made using the Newman-Keuls test. The significance level was set at *p*≤ 0.05. All statistics were two-tailed.

## Results

### Body weight measure

**Fig. 2:**
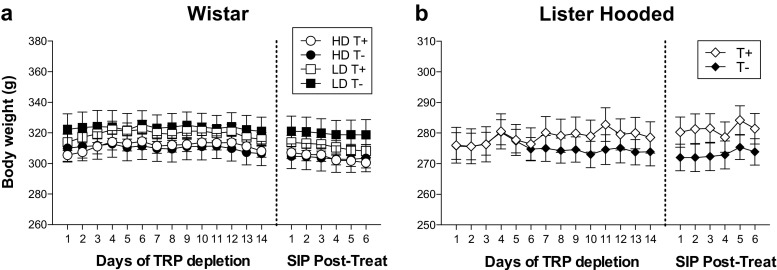
Body weight during 14 days of TRP depletion by diet and six sessions of schedule-induced polydipsia post-treatment for Wistar (**a**) and Lister Hooded rats (**b**). Wistar rats are grouped as TRP non-depleted high drinkers (HD T+), TRP-depleted high drinkers (HD T−), TRP non-depleted low drinkers (LD T+) and TRP-depleted low drinkers (LD T−). Lister Hooded rats are grouped as TRP non-depleted (T+) and TRP-depleted rats (T−). Data are means ± SEM

Figure 2 shows body weight during 14 days of TRP depletion by diet and 6 days of SIP post-treatment in Wistar and Lister Hooded rats. No differences in body weight between T+ and T− groups were found neither in Wistar (treatment effect *F*_1,24_ = 0.09; *p =* 0.761) nor in Lister Hooded rats (treatment effect *F*_1,15_ = 0.496; *p =* 0.492).

### Schedule-induced polydipsia pre-treatment

**Fig. 3:**
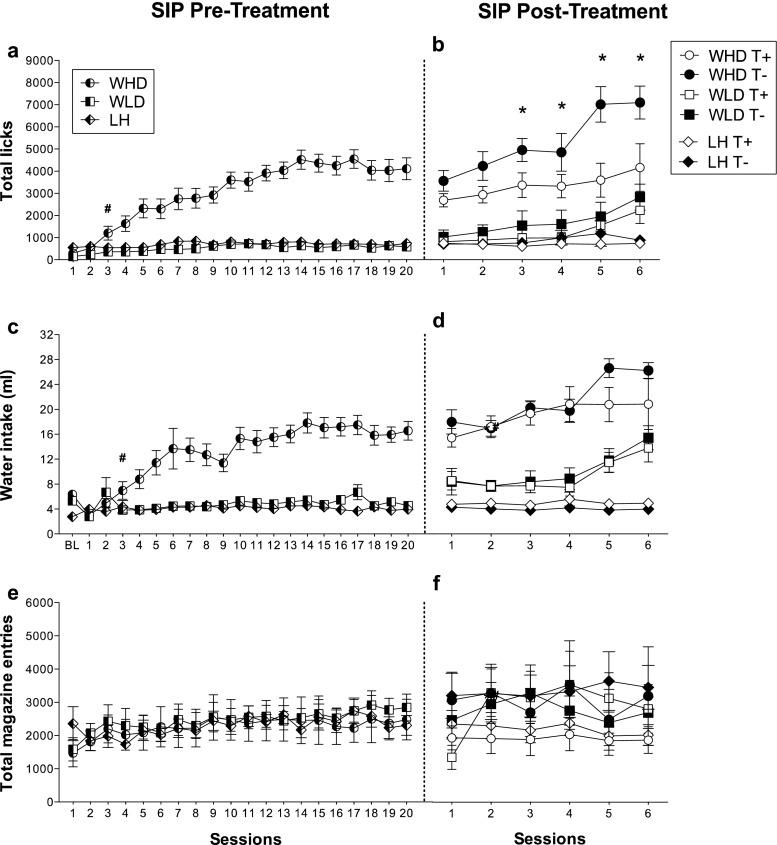
The mean (±SEM) total licks (**a**, **b**), water intake (**c**, **d**) and total magazine entries (**e**, **f**) in FT-60s across 20 sessions of SIP pre-treatment and six sessions of SIP post-treatment for both Wistar and Lister Hooded rats. Rats are grouped in the SIP pre-treatment as high-drinker Wistar rats (WHD), low-drinker Wistar rats (WLD) and Lister Hooded rats (LH). Wistar rats are grouped in the SIP post-treatment as TRP non-depleted high drinkers (WHD T+), TRP-depleted high drinkers (WHD T−), TRP non-depleted low drinkers (WLD T+) and TRP-depleted low drinkers (WLD T−). Lister Hooded rats are grouped in the SIP post-treatment as TRP non-depleted (LH T+) and TRP-depleted rats (LH T−). *Asterisks*: statistical analyses indicate significant differences between HD T+ and HD T−. *Number sign*: statistical analyses indicate significant differences between WHD and WLD from that session onward

Figure 3 shows the mean total licks, water intake and total magazine entries in high-drinker Wistar rats (WHD), low-drinker Wistar rats (WLD) and Lister Hooded (LH) rats on the SIP pre-treatment FT-60s schedule of food presentation. ANOVA revealed significant differences on SIP acquisition between WHD and WLD in total licks (group effect *F*_1,26_ = 71.5; *p* < 0.001) and water intake (group effect *F*_1,26_ = 44.18; *p* < 0.001). SIP session effects were significant in both measures: total licks (*F*_19,494_ = 23.6; *p* < 0.001) and water intake (*F*_19,494_ = 13.03; *p* < 0.001). Interaction between sessions and group was also significant in total licks (*F*_19,494_ = 15.5; *p* < 0.001) and water intake (*F*_19,494_ = 9.24; *p* < 0.001). Post hoc analysis indicated that the FT-60s schedule of food delivery induced different drinking rates across the 20 test sessions in both groups. Differences in total licks between WHD and WLD animals were evident from session 3 (*p =* 0.031) and from session 3 in water intake (*p =* 0.039). WHD animals significantly increased their consumption of water from session 3 (*p =* 0.002) to the end of training, reaching stable levels from session 10. WLD animals did not show a significant increase in their consumption of water across SIP sessions. No interaction effect was found in magazine entries (*F*_19,494_ = 0.56; *p =* 0.933) (Fig. 3e).

No significant differences were found between LH and WLD rats in water intake (strain effect *F*_1,29_ = 1.76; *p =* 0.195), total licks (strain effect *F*_1,29_ = 2.26; *p =* 0.143) and magazine entries (strain effect *F*_1,29_ = 1.56; *p =* 0.696).

### Schedule-induced polydipsia post-treatment

Figure 3 shows the effects of the chronic TRP depletion by diet on Wistar and LH rats on SIP. The TRP depletion in WHD T− rats increased the total number of licks over the days (see Fig. 3b; group × treatment × session effect *F*_5,120_ = 2.46; *p =* 0.037) but did not affect water intake on SIP (see Fig. 3d; group × treatment × session effect *F*_5,120_ = 1.06; *p =* 0.387). Post hoc analysis indicated that the differences on licks between WHD T+ and WHD T− occur from session 3 (*p =* 0.007). WHD T− animals significantly increased their licks rate from session 3 (*p =* 0.021). Differences between WHD and WLD rats remained stable in water intake (group effect *F*_1,24_ = 55.33; *p* < 0.001) and total licks (group effect *F*_1,24_ = 32.44; *p* < 0.001). An increase of total licks was observed in WHD T+ (*p =* 0.020) and WLD T− (*p =* 0.002) on session 6, but these groups remain statistically different from each other (*p =* 0.040). To understand the discrepancy of finding increments in total licks not observed in water intake, we explored licking efficiency in WHD and WLD rats during the six sessions of SIP. Interestingly, we found statistical increases of licking efficiency in WHD T− compared to WHD T+ (treatment × session *F*_5,60_ = 3.283; *p =* 0.011), but no differences were found in WLD T− compared to WLD T+ (treatment × session *F*_5,60_ *=* 0.205*; p =* 0.959) (data not shown). Post hoc analysis revealed increments of licking efficiency in WHD T− compared to WHD T+ from session 2 onwards (*p* < 0.001).

LH T+ and LH T− did not differ in water intake (Fig. 3d; treatment × session effect *F*_5,75_ = 0.353; *p =* 0.879), total number of licks (Fig. 3b; treatment × session effect *F*_5,75_ = 1.013; *p =* 0.416) and licking efficiency (treatment × session *F*_5,75_ = 0.790; *p =* 0.560) (data not shown). TRP depletion by diet did not alter total number of magazine entries neither in the Wistar rats (group × treatment × session: *F*_5,120_ = 1.02; *p =* 0.410) nor in the LH rats (group × treatment × session: *F*_5,75_ = 2.076; *p =* 0.078) (Fig. 3f).

### Spontaneous locomotor activity

**Fig. 4:**
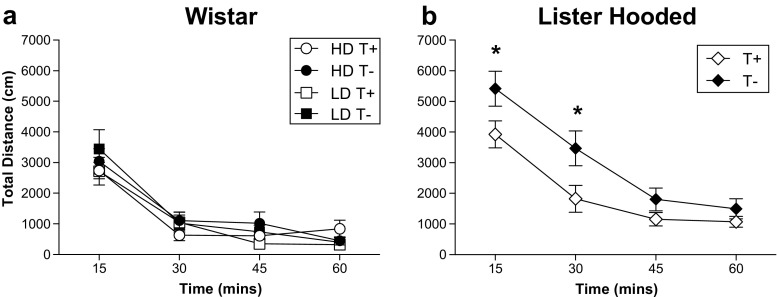
Total distance in four blocks of 15 min for Wistar (**a**) and Lister Hooded rats (**b**). Wistar rats are grouped as TRP non-depleted high drinkers (HD T+), TRP-depleted high drinkers (HD T−), TRP non-depleted low drinkers (LD T+) and TRP-depleted low drinkers (LD T−). Lister Hooded rats are grouped as TRP non-depleted (T+) and TRP-depleted rats (T−). Data are means ± SEM. *Asterisks*: statistical analyses indicate significant differences between T+ and T−

Figure 4 shows locomotor response measured as total distance (cm) in four blocks of 15 min for Wistar and LH rats. TRP depletion by diet increased locomotor response only in the LH rats (treatment × blocks *F*_3.45_ = 3.08; *p =* 0.037). Post hoc analyses revealed that LH T− showed an increased locomotor activity in the first 15 min (*p =* 0.015) and the second 15 min (*p =* 0.008) of the 60-min session compared to LH T+ (Fig. 4b). No effects of the TRP depletion by diet were found in locomotor response in the Wistar rats (treatment × blocks *F*_3,72_ = 1.30; *p =* 0.280), not even considering groups of HD and LD rats (see Fig. 4a; group × treatment × blocks *F*_3.72_ = 0.78; *p =* 0.508). Wistar (*F*_3,72_ = 79.91; *p* < 0.001) and LH rats (*F*_3,45_ = 81.16; *p* < 0.001) decreased the activity over the session significantly.

### Serotonin receptor binding

**Fig. 5:**
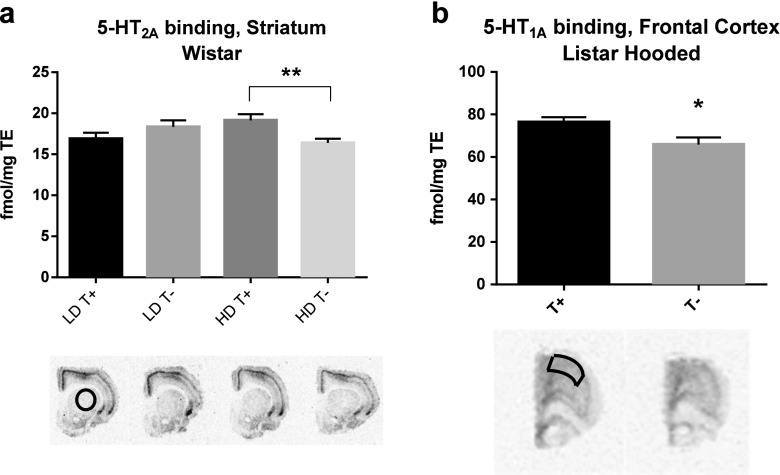
The mean (±SEM) 5-HT_2A_ receptor binding of striatum slices in the Wistar rats (**a**) and 5-HT_1A_ receptor binding of frontal cortex slices in the Lister Hooded rats (**b**). 5-HT_2A_ receptor binding was detected by [^3^H]MDL100907, and 5-HT_1A_ binding was detected by [^3^H]WAY100635. Wistar rats are grouped as TRP non-depleted high drinkers (HD T+), TRP-depleted high drinkers (HD T−), TRP non-depleted low drinkers (LD T+), and TRP-depleted low drinkers (LD T−). Lister Hooded rats are grouped as TRP non-depleted (T+) and TRP-depleted rats (T−). *Asterisks*: statistical analyses indicate significant differences between T+ and T− (*p < 0.05, **p < 0.01)

Table 1 shows mean ± SEM ^3^H-MDL100907 and ^3^H-WAY100635 binding for groups of depleted and non-depleted Wistar and LH groups of rats. For the 5-HT_2A_ receptor in Wistar rats, there was a group × treatment interaction in the striatum for ^3^H-MDL100907 binding (*F*_1,23_ = 8.648; *p =* 0.007) (see Fig. 5a). Post hoc analyses revealed a reduction of 5-HT_2A_ receptor density in WHD T− rats compared to WHD T+ rats (*p =* 0.014). In the frontal cortex, we observed a 10% reduction of 5-HT_2A_ binding in the HD T− compared to HD T+ rats; however, the statistical analysis did not detect statistical differences (group × treatment *F*_1,24_ = 0.990; *p =* 0.330). TRP depletion by diet did not alter 5-HT_2A_ levels of LH T− compared to LH T+ neither in the frontal cortex (*F*_1,16_ = 0.117; *p =* 0.737) nor in the striatum (*F*_1,16_ = 0.066; *p =* 0.801).

**Table 1:** ^3^H-MDL100907 and ^3^H-WAY100635 binding (fmol/mg TE) in the frontal cortex, striatum and hippocampus in all groups of Wistar and Lister Hooded rats

		Wistar				Lister Hooded	
		HD		LD			
		T+	T−	T+	T−	T+	T−
FC	5-HT_2A_	73.16 ± 4.68	65.44 ± 2.87	73.90 ± 4.86	76.39 ± 7.17	102.70 ± 4.59	105.7 ± 7.85
	5-HT_1A_	27.13 ± 0.95	29.07 ± 1.84	26.57 ± 2.07	26.49 ± 2.90	45.44 ± 1.57	37.07 ± 1.00**
Striat	5-HT_2A_	19.13 ± 0.75	16.41 ± 0.49*	16.91 ± 0.73	18.34 ± 0.80	25.51 ± 0.80	25.23 ± 0.70
	5-HT_1A_	10.48 ± 0.33	10.38 ± 0.41	10.03 ± 0.30	9.68 ± 0.28	14.75 ± 0.26	14.41 ± 0.79
Hippo	5-HT_1A_	121.23 ± 4.02	116.6 ± 3.88	111.43 ± 7.42	117.54 ± 8.69	110.73 ± 7.29	96.36 ± 6.33

Data are mean ± SEM. Significant differences between T+ and T− (*p < 0.05, **p < 0.01)

*FC* frontal cortex, *Striat* striatum, *Hippo* hippocampus

The 5-HT_1A_ receptor density in the frontal cortex showed a reduction in LH T− rats compared to LH T+ in terms of ^3^H-WAY100635 binding (*F*_1,16_ = 19.091; *p =* 0.001) (see Fig. 5b), but no differences in density were found in the striatum (*F*_1,16_ *=* 1.80; *p =* 0.677) and the hippocampus (*F*_1,16_ = 2.157; *p =* 0.163). The effect of TRP depletion by diet in the 5-HT_1A_ receptor density observed in LH T− did not occur in the depleted Wistar rats (group × treatment *F*_1,24_ = 0.240; *p =* 0.629). Also, 5-HT_1A_ density was unaffected in the striatum (group × treatment *F*_1,23_ = 0.138; *p =* 0.714) and hippocampus (group × treatment *F*_1,25_ = 1.672; *p =* 0.209) in the depleted Wistar rats.

### Monoamine concentration levels

The chronic TRP depletion by diet significantly reduced 5-HT, 5-HIAA and 5-HIAA/5-HT turnover ratio in the prefrontal cortex (PFC), amygdala and hippocampus in both strains of rats. No interaction effects of group and treatment were found in monoamine concentration levels in the different areas for Wistar rats. In the depleted groups of Wistar rats, 5-HT levels were reduced in the PFC (*F*_1,23_ = 20.86; *p* < 0.001) and hippocampus (*F*_1,24_ = 5.89; *p* < 0.023) (see Table 2), and there was a tendency toward significance in the amygdala (*F*_1,24_ = 3.92; *p =* 0.059). 5-HIAA levels were decreased in the PFC (*F*_1,23_ = 29.52; *p* < 0.001), striatum (*F*_1,22_ = 4.79; *p =* 0.040), amygdala (*F*_1,24_ = 29.79; *p* < 0.001) and hippocampus (*F*_1,24_ = 19.837; *p* < 0.001). In addition, a decreased 5-HIAA/5-HT turnover ratio in all areas was found: PFC (*F*_1,23_ = 16.13; *p* < 0.001), striatum (*F*_1,22_ = 51.90; *p* < 0.001), amygdala (*F*_1,24_ = 52.97; *p* < 0.001), nucleus accumbens (*F*_1,19_ = 19.67; *p* < 0.001) and hippocampus (*F*_1,24_ = 64.45; *p* < 0.001). No significant changes in levels of 5-HT and 5-HIAA were observed in the nucleus accumbens. NE and DA were not significantly affected in any brain regions.

**Table 2:** Monoamine concentration levels (picomole/milligramme of tissue) in the prefrontal cortex, striatum, amygdala, nucleus accumbens and hippocampus in T+ and T− for Wistar rats (*n* = 11–14)

		5-HT	5-HIAA	5-HIAA/5-HT ratio	NE	DA
PFC	T+	1.67 ± 0.15	1.16 ± 0.14	0.70 ± 0.06	1.86 ± 0.14	1.68 ± 0.46
	T−	0.86 ± 0.09**	0.36 ± 0.03**	0.44 ± 0.03**	1.72 ± 0.13	2.87 ± 0.77
Striat	T+	3.56 ± 0.81	4.13 ± 0.88	1.17 ± 0.07	0.14 ± 0.03	130.64 ± 27.47
	T−	3.08 ± 0.47	2.08 ± 0.37*	0.65 ± 0.03**	0.22 ± 0.08	146.89 ± 23.26
Amyg	T+	2.93 ± 0.32	2.36 ± 0.22	0.83 ± 0.04	1.10 ± 0.21	18.89 ± 3.57
	T−	*2.16 ± 0.21* ^#^	1.01 ± 0.09**	0.48 ± 0.02**	1.47 ± 0.25	20.23 ± 2.83
NAc	T+	2.78 ± 0.75	2.50 ± 0.69	0.90 ± 0.02	2.50 ± 0.55	41.04 ± 13.00
	T−	2.61 ± 1.22	1.45 ± 0.59	0.62 ± 0.06**	5.73 ± 2.58	44.67 ± 20.58
Hippo	T+	1.23 ± 0.15	1.70 ± 0.22	1.38 ± 0.03	1.97 ± 0.20	1.11 ± 0.19
	T−	0.78 ± 0.10*	*0.67* ± 0.05**	0.91 ± 0.04**	1.56 ± 0.11	1.72 ± 0.25

Data are mean ± SEM. Significant differences between T+ and T− (*p < 0.05, **p < 0.01, ^#^ *p =* 0.059)

*PFC* prefrontal cortex, *NAc* nucleus accumbens, *Amyg* amygdala, *Striat* striatum, *Hippo* hippocampus

In the depleted group of LH rats, 5-HT levels were reduced in the PFC (*F*_1,15_ = 33.43; *p* < 0.001), striatum (*F*_1,14_ = 16.82; *p* < 0.001), amygdala (*F*_1,14_ = 6.63; *p =* 0.022) and hippocampus (*F*_1,15_ = 9.10; *p =* 0.009) (see Table 3). 5-HIAA levels and 5-HIAA/5-HT turnover ratio decreased in the PFC (*F*_1,13_ = 38.89; *p* < 0.001; *F*_1,13_ = 10.25; *p =* 0.007), striatum (*F*_1,14_ = 21.82; *p* < 0.001; *F*_1,14_ = 14.45; *p =* 0.002), amygdala (*F*_1,14_ = 31.85; *p* < 0.001; *F*_1,14_ = 40.83; *p* < 0.001), nucleus accumbens (*F*_1,11_ = 17.65; *p* < 0.001; *F*_1,11_ = 20.56; *p* < 0.001) and hippocampus (*F*_1,15_ = 32.38; *p* < 0.001; *F*_1,15_ = 56.49; *p* < 0.001). Besides, there was a compensatory increase in DA in nucleus accumbens (*F*_1,11_ = 8.28; *p =* 0.015) and a decrease in NE in PFC (*F*_1,15_ = 6.42; *p =* 0.023); DA and NE were not affected in other areas.

**Table 3:** Monoamine concentration levels (picomole/milligramme of tissue) in the prefrontal cortex, striatum, amygdala, nucleus accumbens and hippocampus in T+ and T− for Lister Hooded rats (*n* = 4–9)

		5-HT	5-HIAA	5-HIAA/5-HT ratio	NE	DA
PFC	T+	1.77 ± 0.09	1.02 ± 0.09	0.56 ± 0.04	1.62 ± 0.09	3.89 ± 1.08
	T−	0.99 ± 0.11**	0.04 ± 0.07**	0.35 ± 0.07**	1.31 ± 0.08*	2.34 ± 0.72
Striat	T+	2.20 ± 0.27	1.98 ± 0.31	0.88 ± 0.04	0.25 ± 0.07	79.35 ± 11.61
	T−	0.92 ± 0.15**	0.52 ± 0.06**	0.60 ± 0.06**	0.22 ± 0.08	83.19 ± 7.21
Amyg	T+	2.81 ± 0.32	2.12 ± 0.21	0.78 ± 0.05	1.38 ± 0.28	12.94 ± 2.56
	T−	1.75 ± 0.21*	0.68 ± 0.09**	0.39 ± 0.02**	1.13 ± 0.34	20.18 ± 3.28
NAc	T+	2.74 ± 0.31	2.34 ± 0.19	0.90 ± 0.06	8.01 ± 1.98	23.14 ± 4.36
	T−	2.09 ± 0.22	1.01 ± 0.15**	0.48 ± 0.02**	3.40 ± 1.96	48.99 ± 9.52*
Hippo	T+	1,45 ± 0.19	2.17 ± 0.24	1.54 ± 0.07	2.60 ± 0.23	4.95 ± 1.71
	T−	0.79 ± 0.07**	0.64 ± 0.07**	0.81 ± 0.06**	3.05 ± 0.62	9.45 ± 3.46

Data are mean ± SEM. Significant differences between T+ and T− (*p < 0.05, **p < 0.01)

*PFC* prefrontal cortex, *NAc* nucleus accumbens, *Amyg* amygdala, *Striat* striatum, *Hippo* hippocampus

## Discussion

The present study has shown the effects of chronic TRP depletion by diet in two strains of rats: Wistar and LH. Before TRP depletion by diet, we examined between-strain differences in the model of compulsive behaviour, SIP, and we found in the Wistar strain two groups of rats based on their drinking behaviour, HD and LD, while the LH strain did not show acquisition of compulsive drinking. After the chronic TRP depletion by diet, the TRP-depleted HD group of Wistar rats increased their compulsive drinking based on the total number of licks, but no changes in drinking behaviour were observed in either the LD Wistar or LH rats. Conversely, TRP depletion produced an increase in spontaneous locomotor activity only in LH rats, while the Wistar rats were unaffected. A reduction of striatal 5-HT_2A_ receptor binding was observed in depleted HD Wistar rats compared to non-depleted HD Wistar rats, while depleted LD Wistar rats were not affected by the TRP manipulation. On the contrary, depleted LH rats showed reduced binding of the 5-HT_1A_ receptor in the frontal cortex. Monoamine measures confirmed that 5-HT, 5-HIAA and the 5-HIAA/5-HT ratio were depleted in different brain regions in both Wistar and LH rats. These results will be further discussed in terms of the relationship between serotonin and inhibitory control.

### Acquisition of schedule-induced polydipsia and strain differences

In the SIP procedure, the exposure of the Wistar strain to an FT-60s schedule of food delivery differentiated two populations based on the amount of drinking: high and low drinkers. The HD Wistar rats showed an increased volume of water intake and number of licks from session 3 compared to the LD Wistar rats. These results confirm previous studies in which consistent individual differences are found (for review, see Moreno and Flores 2012). However, the LH rats did not show an acquisition of SIP. This study is the first to evaluate strain differences between Wistar and LH rats in SIP acquisition and the development of SIP drinking. Regarding strain differences, LH rats compared to Wistar rats show a higher inhibitory control measured by less anticipatory responses in the 3-CSRT task and less food hoarding behaviour (Broersen and Uylings 1999). Other strains exhibiting inhibitory control deficits have shown increased SIP behaviour. For instance, spontaneously hypertensive rats, characterized as hyperactive and impulsive in terms of exacerbated sensitivity to delay of reinforcement, displayed increased drinking in SIP compared to Wistar–Kyoto rats (Ibias and Pellón 2011), as well as two rat lines selectively bred for high ethanol preference compared to their non-preferring counterparts (Gilpin et al. 2008). Moreover, the selective breeding of Roman high- (RHA) and low-avoidance (RLA) rats for rapid vs. extremely poor acquisition of active avoidance behaviour in a shuttle box resulted in two phenotypes that present differences in SIP acquisition (Moreno et al. 2010). RHA rats, which show traits such as higher novelty seeking, susceptibility to addictive drugs and impulse behaviours in the delay-discounting task and five-choice serial reaction time (5-CSRT) task (Escorihuela et al. 1999; Fattore et al. 2009; Moreno et al. 2010), also display increased SIP acquisition compared to RLA rats. Thus, SIP seems to be sensitive in distinguishing phenotypes of rats that have shown deficits in inhibitory control responses in different tasks of impulsivity/compulsivity, indicating a lack of inhibitory control as the main characteristic involved in the compulsive drinking in SIP (Moreno and Flores 2012).

### Effect of chronic tryptophan-deficient diet on schedule-induced polydipsia and possible mechanisms

Chronic TRP-deficient diet exposure increased the total number of licks in the HD Wistar rats without affecting the amount of water drunk on SIP. We found similar observations in our laboratory of an increase in total licks on SIP after 6 months of an acute chlorpyrifos (CPF) administration (Cardona et al. 2006, 2011). There is evidence that long-term CPF intoxication affects the serotonergic system (Chen et al. 2011; Moreno et al. 2008), possibly by inducing TRP hydroxylase, the rate-limiting enzyme for 5-HT biosynthesis, and suppressing expression of 5-HT transporter genes (Slotkin and Seidler 2008). Therefore, a disruption in serotonin levels may be the underlying mechanism for the increased total licks observed on SIP. On the other hand, the increase in licks is task-dependent because groups differ from session 3 onward and not from session 1. The effect of increasing the number of licks without affecting the amount of water intake, also observed by Cardona et al. (2006, 2011), may suggest a change of the drinking behaviour understood as an expression of compulsivity. In this sense, lick efficiency analyses showed differences in HD Wistar rats due to the TRP depletion by diet. This result could be interpreted as an increase in the stereotypic/compulsive manner of drinking by depleted HD rats, and this increase is not due to motor problems since depleted LD Wistar and LH rats did not differ in lick efficiency. The specific effect of the chronic TRP depletion increasing total licks and licking efficiency in HD Wistar rats but not in LD Wistar rats indicates a vulnerability of the HD group to compulsive symptoms and an implication of the serotonergic system mediating this vulnerability.

Only a few studies have tested the effect of acute TRP depletion in OCD patients, showing not significant increases of obsessions or compulsions according to the scores of the Yale Brown Obsessive Compulsive Scale at rest or following symptom provocation (Barr et al. 1994; Berney et al. 2006). However, taking into account studies using neuropsychological tasks instead of questionnaires, patients with psychopathologies from the impulsive-compulsive spectrum seem to aggravate their symptoms when exposed to ATD. For instance, ATD increased omissions in the continuous performance task (Mette et al. 2013; Zepf et al. 2010) and aggressive behaviour in ADHD patients (Kötting et al. 2013; Stadler et al. 2007; Zepf et al. 2008, 2009; Zimmermann et al. 2012). Interestingly, ATD impaired go/no-go performance (LeMarquand et al. 1999) and stop signal reaction time (Crean et al. 2002) in healthy men with family history of alcoholism and also increased commission errors in the go/no-go task in an aggressive subgroup of people with ADHD (LeMarquand et al. 1998). These findings suggest that ATD may reveal vulnerable 5-HT systems in certain populations at risk of impulse control disorders (Faulkner and Deakin 2014), though it is still unknown precisely which receptor subtypes may lay behind this vulnerability.

In our study, we found a reduction of striatal 5-HT_2A_ receptor density in TRP-depleted HD Wistar rats compared to non-depleted HD Wistar rats. No differences were obtained in the LD Wistar or LH rats. Alterations of 5-HT_2A_ receptor levels in 5-HT depletion studies are controversial. Several studies report upregulation of this receptor subtype in the hippocampus and frontal cortex (Franklin et al. 2012; Heal et al. 1985; Seeman et al. 1980), while other studies do not observe any difference (Blackshear et al. 1981; Conn and Sanders-Bush 1986; Fischette et al. 1987). In support of our findings, Licht et al. (2009) found that 5-HT_2A_ receptor binding was markedly reduced in striatum and prefrontal cortex regions after 5-HT depletion. Barlow et al. (2015) had similar findings regarding the 5-HT_2A_ receptor reductions and low levels of 5-HT in the orbitofrontal cortex in perseverative rats in the reversal learning task. They furthermore reported differences in gene expression levels of the MAO-A and MAO-B enzymes. They suggest that decreased MAO activity in the DRN resulted in reduced 5-HT breakdown and consequently increased autoinhibition of 5-HT neurons by somatodendritic 5-HT receptors (Barlow et al. 2015; Liu et al. 2005).

The specific downregulation of the striatal 5HT_2A_ receptor in HD but not LD rats by manipulation of the central 5-HT system reveals a specific role of the 5-HT_2A_ receptor system in the observed increase in compulsive drinking on SIP. Evidence from animal and human studies underlies a key role of the 5-HT_2A/C_ receptors in compulsive symptoms (Fineberg et al. 2010, 2011). Activation of prefrontal 5-HT_2A_ receptors has been proposed to underpin the anticompulsive effect of SSRIs (Dannon et al. 2000; for a review, see El Mansari and Blier 2006; Westenberg et al. 2007). Second-generation antipsychotics may exacerbate compulsive behaviours in patients with schizophrenia and proposed to be through the potent 5-HT_2A_ antagonism (Poyurovsky et al. 2008). In fact, activation of the 5-HT_2A/C_ by DOI reduces compulsive drinking on SIP, and this reduction is blocked by the 5-HT_2A_ receptor antagonists ketanserin and M100907, but not by the 5-HT_2C_ receptor antagonist SB242084, indicating that the 5-HT_2A_ receptor mediates the anticompulsive effect of DOI on SIP (Navarro et al. 2015). Moreover, systemic administration of M100907 in rats impairs spatial reversal learning, increasing perseverative responses (Boulougouris et al. 2008). Alterations in 5-HT_2A_ receptor levels have also been observed in Roman high-avoidance (RHA) rats (Klein et al. 2014), an inbred strain characterized by a compulsive drinking profile on SIP, impulsivity on the delay-discounting task and poor inhibitory control in the 5-CSRT task (Moreno et al. 2010). In humans, neuroimaging studies have also linked differences in 5-HT_2A_ receptor levels to the development of compulsive spectrum disorders. Positron emission tomography (PET) studies in drug-naive OCD patients reveal a reduction in frontal cortex serotonin 5-HT_2A_ receptor availability (Perani et al. 2008), with specific correlations between serotonin 5-HT_2A_ receptor availability in the orbitofrontal cortex and age of onset of the disorder (Simpson et al. 2011).

Little is known regarding the contribution of the striatal serotonin receptor subtypes to cognitive function. In the striatum, 5-HT receptors modulate the activity of DA, GABA and glutamate neurotransmission and output regions of the basal ganglia (Nicholson and Brotchie 2002), suggesting a role of the 5-HT system in regulating action selection and motor control (Di Matteo et al. 2008). More studies are needed to evaluate the role of the striatal 5-HT_2A_ receptor in the impulsive-compulsive spectrum disorders.

### Effect of chronic tryptophan-deficient diet on spontaneous locomotor activity and possible mechanisms

The diet-induced chronic TRP depletion resulted in the LH strain in an increase in spontaneous locomotor activity not observed in the Wistar strain. Contrary to our results, previous studies with Wistar rats reported increases in locomotor activity after a 5-week exposure to a TRP-deficient diet (Vergnes and Kempf 1981). Possibly, the effect of TRP depletion by diet on locomotor activity depends on the severity of 5-HT reductions, based on the observations of greater global reductions of 5-HT and its metabolite in LH but not in Wistar rats in our study. In fact, the increases of locomotor activity in Vergnes and Kempf’s study (1981) were found after a period of 5-week exposure to a TRP-free diet, in which 75% of reductions in brain 5-HT levels were observed. However, we have carried out an exposure of 2 weeks that is similar to the chronic TRP treatment in Fadda et al.’s study (2000), in which 35–45% of 5-HT reductions were found. Moreover, central 5-HT depletion by administration of 5,7-dihydroxytryptamine, an invasive neurotoxic method that drastically reduces 5-HT levels, reported an increase in locomotor activity in LH rats (Eagle et al. 2008). On the other hand, our results confirm previous observations of no differences in locomotor activity between the HD and LD Wistar rats (Moreno et al. 2010).

Besides this effect, depleted LH rats showed a downregulation of prefrontal 5-HT_1A_ receptor that was not observed in the depleted Wistar rats. This is interesting as 5-HT_1A_ receptors seem to be less susceptible to changes in serotonergic tonus compared to other 5-HT receptor subtypes, based on several studies of 5,7-DHT lesions (Berendsen et al. 1991; Frazer and Hensler 1990; Hensler et al. 1991; Miquel et al. 1992; Verge et al. 1986). However, Kawai et al. (1994) proposed a downregulation of 5-HT_1A_ receptors in the frontal cortex as a homeostatic adaptive change in response to chronic TRP deprivation.

### Effect of chronic tryptophan-deficient diet on monoaminergic concentration levels

Chronic TRP depletion was effective in reducing the levels of 5-HIAA/5-HT turnover ratio in prefrontal cortex, striatum, amygdala, nucleus accumbens and hippocampus in both strains of rats. In addition, the serotonin metabolite 5-HIAA was reduced in all cases with the exception of nucleus accumbens of the Wistar rats. More variability was found when exploring significant reductions of 5-HT, which was effectively decreased in all areas but not in the striatum of the Wistar rats and the nucleus accumbens of both strains. Previous studies have reported similar findings regarding significant reductions of serotonin and its metabolite in prefrontal cortex and hippocampus when challenging rats to a TRP depletion by diet (Cahir et al. 2007; Franklin et al. 2012; Koot et al. 2012). However, there were strain differences, as the LH rats showed additional changes in other monoamines such as DA, which was increased in the nucleus accumbens, and NE, which was decreased in the prefrontal cortex, not observed in the Wistar rats. Alterations of DA and NA when depleting chronically TRP by diet were also observed by Koot et al. (2012) in Wistar rats, indicating that this non-invasive depleting method of 5-HT may possibly lead to alterations in other brain neurotransmitter systems.

In conclusion, the primary findings of the present study highlight the contribution of serotonergic mechanisms in the compulsive drinking behaviour of rats on SIP, in which the serotonin depletion by chronic exposure to a TRP-free diet increased compulsive licking in HD Wistar rats compared to LD Wistar and LH rats on SIP. Moreover, the TRP depletion by diet produced a modulation of 5-HT_1A_ and 5-HT_2A_ receptor subtypes. The depleted HD Wistar rats showed 5-HT_2A_ receptor reductions in the striatum, which may underlie the increases in licking on SIP. Changes in the 5-HT_2A_ receptor subtype may represent a good potential biomarker for the vulnerability to compulsive spectrum disorders and a new target in the development of new therapeutic strategies.
